# When Fairness Backfires: How Organizational Justice Amplifies the Strain of Leader–Member Exchange Ambivalence

**DOI:** 10.3390/bs15101424

**Published:** 2025-10-20

**Authors:** Rui Ma, Haiqing Bai, Jin Cheng, Huichi Qian

**Affiliations:** 1 School of Management, Xiamen University, Xiamen 361005, China; rui.ma.edu@xmu.edu.cn (R.M.); qianhuichi@stu.xmu.edu.cn (H.Q.); 2 School of Journalism and Communication, Xiamen University, Xiamen 361005, China; haiqing.bai@xmu.edu.cn

**Keywords:** leader–member exchange ambivalence, interactional justice, workplace sense of control, emotional exhaustion

## Abstract

This research examines how leader–member exchange ambivalence (LMXA) affects employee emotional exhaustion. It investigates the mediating role of workplace sense of control and the moderating effects of interactional justice. Based on Fairness Heuristic Theory, this research proposes that high organizational justice amplifies rather than buffers LMXA’s detrimental effects due to violated fairness expectations. Data from 511 Chinese employees were collected through a two-phase survey and analyzed using moderated mediation analysis. Results show that LMXA positively relates to emotional exhaustion through reduced workplace sense of control, and interactional justice strengthens this indirect effect. The negative impact of LMXA on workplace sense of control increases when justice levels are high, consequently increasing emotional exhaustion. These findings reveal a paradoxical effect of organizational justice, challenging assumptions about its universally positive function. This research contributes by demonstrating that fair organizational systems could backfire when combined with inconsistent leadership. The findings provide insights into how employees manage relational uncertainty and highlight the importance of leadership consistency in organizations.

## 1. Introduction

The relationship between leaders and their employees serves as a foundation of workplace dynamics, significantly shaping employee attitudes, behaviors, and overall well-being (Dulebohn et al., 2012). Leader–member exchange (LMX) theory has long dominated this research area, typically characterizing leader–follower relationships as either high-quality exchanges built on trust and mutual respect, or low-quality, formal interactions (Henderson & Jeong, 2024; Martin et al., 2018). While this framework has provided valuable insights, it neglects a common psychological reality in the workplace. Many employees have mixed feelings toward their leaders, experiencing both positive and negative emotions simultaneously (Han & Sears, 2024; Methot et al., 2017). An individual might, for instance, respect a manager’s decision-making while simultaneously feeling frustrated by their inconsistent communication and support. These conflicting positive and negative emotions create leader–member exchange ambivalence (LMXA), leaving employees in a state of relational uncertainty and psychological stress (Lee et al., 2019).

The unpredictable nature of LMXA is a significant challenge, with a growing body of research demonstrating its link to detrimental outcomes like job anxiety and burnout (Han & Sears, 2024; Huang et al., 2022). However, a critical gap remains. While scholars have stated that LMXA is harmful (Liu & Liu, 2024; Setthakorn, 2023; Lee et al., 2019), the underlying psychological process that explains how this relational uncertainty translates into emotional strain is still underdeveloped (Han & Sears, 2024). This research argues that the lack of employee’s workplace sense of control could act as a mediating psychological effect between LMXA and psychological strain. Sense of control fundamentally relies on a predictable environment where actions lead to foreseeable outcomes (Skinner, 1996). LMXA, with its inherent inconsistency, directly attacks this foundation. When a leader’s behavior unpredictably alternates between support and criticism, employees may lose the ability to predict the consequences of their efforts. The ambivalent leader–follower interaction directly damages employees’ belief that they can influence their work environment (Infurna & Infurna, 2017). This loss of control is an influential stressor, making it a prime factor for the key mechanism linking LMXA to emotional exhaustion (Maslach et al., 2001).

Beyond understanding how LMXA leads to psychological strain, it is equally important to identify the conditions under which its effects become most distinct. At first glance, a fair and just organizational context might be expected to shield employees from harmful ambivalent relationships. When an organization consistently treats its members with respect and dignity (i.e., demonstrates high interactional justice), this should theoretically provide relational stability when encountering an unpredictable leader (Holtz, 2013; J. A. Colquitt et al., 2013a). Yet this research advances a counterintuitive proposition that rather than buffering against harm, a high-justice environment may amplify the negative consequences of LMXA. This paradoxical prediction draws on Fairness Heuristic Theory (FHT).

FHT suggests that under conditions of uncertainty, individuals rely on fairness perceptions as a mental shortcut to evaluate authority figures and anticipate future treatment (Lind, 2001). A context characterized by high interactional justice does more than provide a neutral environment. It signals that leaders are trustworthy and will behave consistently and ethically (Van den Bos, 2005). Through this heuristic process, employees develop strong and positive expectations about how they will be treated. Prior research has demonstrated the power of such fairness perceptions. When people perceive fairness in their interactions with authorities, they become more resilient to various outcomes (Matta et al., 2017; J. A. Colquitt et al., 2013b). This occurs since fairness serves as an indicator of reliability and respect. However, this mechanism may lead to negative effects and heighten psychological strain.

When a leader behaves ambivalently within a high interactional justice context, their actions create a significant violation of expected interpersonal fairness. This violation is particularly significant as both LMXA and interactional justice operate at the relational level. In contrast to other types of organizational justice (distributive justice and procedural justice), interactional justice concerns the quality of interpersonal treatment employees receive from their leaders, including respect, dignity, and honesty in daily interactions (J. A. Colquitt et al., 2001). High interactional justice environments create strong expectations for consistent interpersonal treatment, yet LMXA introduces ambivalence into the relational space. Research shows that employees are especially sensitive to such interpersonal inconsistency when their fairness perceptions are strong (Qin et al., 2015). The contrast between organizational fairness signals and leader ambivalence intensifies psychological strain by undermining established interpersonal trust (Niedeggen et al., 2019). Thus, rather than buffering against psychological strains, interactional justice amplifies the negative impact of LMXA.

**Figure 1:**
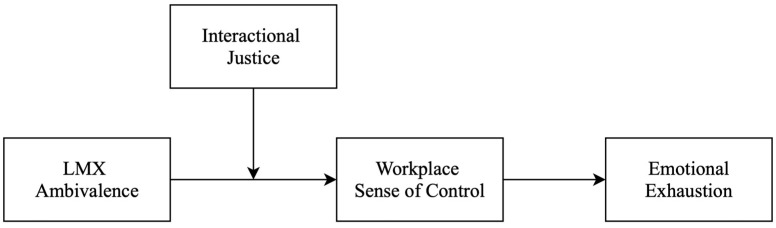
Research model.

To address this theoretical paradox, this research develops and tests a moderated mediation model (Figure 1). The model examines whether LMXA increases emotional exhaustion through diminished workplace sense of control. More importantly, it investigates whether high interactional justice amplifies this indirect effect.

This research makes two key contributions. First, by identifying workplace sense of control as the mediating mechanism, it reveals a psychological pathway through which LMXA generates emotional exhaustion. Second, by applying FHT to the LMXA context, it challenges the assumption that organizational fairness uniformly protects employees. The findings demonstrate that interactional justice can amplify rather than buffer the harmful effects of leader ambivalence. Building on this foundation, the literature review examines the key constructs and develops specific hypotheses.

## 2. Conceptual Background and Hypothesis Development

### 2.1. Leader–Member Exchange Ambivalence (LMXA) and Emotional Exhaustion

Leader–member exchange (LMX) theory conceptualizes leader–follower relationships as differentiated exchanges that vary in quality (Graen & Uhl-Bien, 1995). For decades, research has long established that high-quality LMX relationships benefit employees. These relationships, characterized by mutual trust, respect, and effective communication, lead to higher job satisfaction, enhanced citizenship behavior, and improved performance (Yang et al., 2023; Kim & Koo, 2017). Conversely, low-quality LMX relationships are predominantly transactional in nature, characterized by interpersonal distance, formal interactions, and adherence to contractual obligations rather than mutual investment (Yang et al., 2023; Cropanzano et al., 2017; Dulebohn et al., 2012). However, recent research challenges a purely positive or negative view of workplace relationship, as many employees hold conflicting and mixed feelings toward their leader (Han & Sears, 2024; Zhao & Zhou, 2021; Methot et al., 2017; Pratt & Dirks, 2017).

To capture this complexity, the concept of LMX ambivalence (LMXA) was introduced. Ambivalent relationships contain both positive and negative elements simultaneously, which means in a leader–member interaction may include trust and distrust, support and neglect, or appreciation and frustration (Lee et al., 2019). The conflicting behaviors create psychological tension for employees (ibid). For instance, an employee might admire their leader’s strategic vision (a positive element) while simultaneously feeling undermined by their inconsistent feedback, micromanagement, or lack of personal support (a negative element) (Gottfredson & Aguinis, 2017). This creates a complex and unpredictable relational dynamic that is qualitatively distinct from relationships that are consistently poor or consistently strong (Han & Sears, 2024; Methot et al., 2017).

The paradoxical nature of LMXA represents a significant form of relational uncertainty in contemporary workplace dynamics. Lee et al. (2019) empirically validated LMXA as a distinct construct, demonstrating that it differs from average LMX quality measures. Their findings reveal that LMXA predicts unique variance in performance outcomes, which occurs even after controlling for both average LMX quality and LMX variability. These results confirm that relational ambivalence is a psychologically meaningful state with tangible organizational consequences (Lee et al., 2019). This theoretical advancement is particularly significant in modern organizational contexts, where leaders increasingly face competing pressures, resource constraints, and conflicting demands (Liu & Liu, 2024; Setthakorn, 2023). These challenges make maintaining consistently high-quality exchanges more difficult (Liu & Liu, 2024; Gottfredson & Aguinis, 2017).

Employees in such ambivalent relationships may struggle to predict how their leader will behave in the next interaction or whether organizational rules will be applied consistently, creating a state of cognitive conflict and social ambiguity (Rothman & Melwani, 2017). Empirical research demonstrates that LMXA is detrimental to employee well-being. It connects to negative outcomes such as increased job anxiety, emotional exhaustion, and ego depletion, which ultimately lead to overall poor well-being (Liu & Liu, 2024; Han & Sears, 2024; Huang et al., 2022). The ambivalence consumes valuable mental resources, undermines the satisfaction of basic psychological needs, and ultimately increases the risk of emotional exhaustion and burnout (Rothman et al., 2017). Therefore, the first hypothesis is proposed:

**H1.**  *Leader–Member Exchange Ambivalence (LMXA) is positively associated with employees’ emotional exhaustion.*

The direct association between LMXA and emotional strain is increasingly established in the research field (Liu & Liu, 2024; Huang et al., 2022). However, the underlying psychological mechanisms require further exploration. Specifically, how relational ambivalence translates into adverse outcomes remains unclear (Han & Sears, 2024). This research addresses this gap by investigating workplace sense of control as a key mediator. Employees’ perceived ability to influence their work environment plays a crucial role in workplace well-being (Infurna & Infurna, 2017). The following analysis examines how LMXA’s relational uncertainty reduces control perceptions and leads to emotional exhaustion.

### 2.2. The Mediating Role of Workplace Sense of Control

The concept of workplace sense of control builds on the broader psychological construct of sense of control. The original concept of sense of control refers to individuals’ general beliefs about their ability to influence important outcomes in their lives (Skinner, 1996; Spector, 1986). Workplace sense of control represents individual’s perceived capability to influence workplace events, decisions, and outcomes (Galvin et al., 2018; Burke et al., 1993). It is a fundamental psychological resource that enables employees to effectively navigate workplace challenges and stressors (Skinner, 1996). In organizational contexts, workplace sense of control is associated with positive employee outcomes (Nauman et al., 2020). For instance, workplace sense of control has been demonstrated to mediate the detrimental effects of role ambiguity, job insecurity, and supervisor incivility on employee strain and well-being (Nauman et al., 2020; Meier & Gross, 2015; O’driscoll & Beehr, 2000). When individuals perceive a strong sense of control at work, they tend to present greater confidence, proactivity, and resilience (Bandura, 2006). Conversely, perceived lack of control could engender feelings of helplessness and powerlessness, leading to reduced well-being, increased stress, workplace disengagement, and ultimately, emotional exhaustion (Infurna & Infurna, 2017).

LMXA could damage an employee’s workplace sense of control through its unpredictability. In high-quality LMX relationships, employees may attribute leader support to goodwill and trust (Martin et al., 2016). This positive dynamic could enhance employees’ workplace sense of control, as leaders provide consistent support, resources, and predictable feedback (Kim & Koo, 2017; Graen & Uhl-Bien, 1995). In contrast, in low-quality LMX relationships, employees may attribute neglect to disinterest or transactional motives (Yang et al., 2023). In both cases, employees have stable and predictable expectations.

Ambivalent relationships present a different challenge, as employees need to adjust conflicting thoughts: Is the leader trustworthy or not? Does the leader intend to support or to undermine? The constant doubt generates emotional complexity (Rothman & Melwani, 2017), which depletes cognitive resources and prevents employees from developing a clear workplace sense of control. These mixed signals may confuse an employee’s understanding of the work environment and damage their confidence in affecting outcomes. For instance, when a supervisor provides encouragement on one occasion but then undermines the employee’s work on another, the employee loses the ability to anticipate future interactions (Han & Sears, 2024; Lee et al., 2019).

It is important to distinguish workplace sense of control from related constructs to clarify its unique mediating role. Self-efficacy refers to beliefs about one’s capability to perform tasks (Bandura, 2012). However, LMXA does not primarily undermine competence beliefs but rather erodes confidence in whether applying those skills will lead to predictable outcomes. Similarly, psychological safety concerns the perceived consequences of taking interpersonal risks (Edmondson & Lei, 2014). While LMXA may harm psychological safety, its impact extends beyond social interactions to encompass broader work outcomes. Workplace sense of control uniquely captures this perception of environmental predictability and controllability, making it the most appropriate mediator for understanding how LMXA generates psychological strain.

The leader–follower relationship serves as a primary source of workplace structure and predictability. Leaders shape employees’ understanding of their work environment by providing clear expectations, consistent feedback, and reliable information (Ashford et al., 2016). This consistency enables employees to form stable cognitive maps and feel confident that their actions will lead to predictable outcomes. However, an ambivalent leader undermines this stabilizing function by becoming a source of unpredictability (Lee et al., 2019). Mixed signals such as alternating praise and criticism create an unmanageable state of uncertainty that directly erodes workplace sense of control.

Furthermore, deciphering inconsistent leader behavior forces employees into a reactive stance (Rothman et al., 2017). Rather than executing tasks with confidence, employees must constantly monitor and adjust to shifting supervisory attitudes. This defensive posture consumes cognitive resources and diminishes feelings of agency over work (Bandura, 2006). Thus, by creating relational unpredictability, ambivalent leader behaviors directly damage employees’ sense of control.

Therefore, ambivalent leader behaviors create relational unpredictability, deprive employees of stable expectations, and violate consistency, all of which damage employees’ perceptions of control:

**H2.**  *Leader–Member Exchange Ambivalence (LMXA) is negatively associated with employees’ workplace sense of control.*

A reduced workplace sense of control is a critical predictor of emotional exhaustion. Emotional exhaustion, the core component of job burnout, reflects a psychological state of being emotionally overloaded and drained by one’s work (Maslach et al., 2001). A strong workplace sense of control is a crucial psychological resource that builds resilience against such strain. When employees perceive they could influence their work outcomes, they demonstrate greater well-being (Infurna & Infurna, 2017). Maslach and Leiter (2016) found that healthcare workers, teachers, and other service professionals who maintained a strong workplace sense of control showed greater resilience to emotional exhaustion, even when facing high job demands. In contrast, the loss of control is a key psychological mechanism that translates workplace stressors into strain (Nauman et al., 2020). Without a sense of control, they are forced to reactively endure pressures rather than proactively manage them (Aronsson et al., 2017). This constant state of reactive coping gradually drains an individual’s energy and increases emotional exhaustion (H. Chen et al., 2023; Bandura, 2006).

This research suggests that workplace sense of control plays as a mediating mechanism between LMXA and emotional exhaustion. First, as established in the preceding arguments, the relational unpredictability inherent in LMXA damages an employee’s workplace sense of control. Second, the reduced sense of control makes employees become psychologically depleted and unable to cope with further work demands, which in turn leads to emotional exhaustion. This leads to the following mediation hypothesis:

**H3.**  *Workplace sense of control mediates the relationship between Leader–Member Exchange Ambivalence (LMXA) and emotional exhaustion.*

### 2.3. The Moderating Role of Interactional Justice

After identifying workplace sense of control as the key psychological mechanism, it is critical to consider the broader organizational context that might strengthen or weaken this indirect effect. Organizational justice, which refers to employees’ perceptions of fairness at work, is a crucial contextual factor. Justice is typically understood through three primary dimensions: distributive justice (the perceived fairness of outcomes), procedural justice (the perceived fairness of processes), and interactional justice (the perceived fairness of interpersonal treatment) (J. A. Colquitt, 2001). This research argues that interactional justice is the most theoretically relevant moderator for understanding the effects of LMXA.

The rationale for this focus on interactional justice is grounded in the principle of correspondence. LMXA is an inherently relational stressor arising from the quality of daily exchanges between employees and their leaders (Lee et al., 2019). Interactional justice, which concerns the respect, dignity, and interpersonal treatment employees receive from their supervisors, operates at the same relational level (J. A. Colquitt et al., 2013a). It is therefore the most direct contextual factor shaping how employees interpret leader ambivalent behavior.

In contrast, the other two justice dimensions are less relevant to this relational dynamic. Distributive justice is conceptually distant. An employee may receive fair pay or rewards while still experiencing inconsistent treatment from their leader (Rea et al., 2021; Brown et al., 2023). Similarly, procedural justice pertains to formal organizational rules and processes (Qi et al., 2023; Van den Bos, 2020). An organization can maintain fair procedures, yet this does not prevent a leader from behaving ambivalently in daily interactions. Therefore, to maintain theoretical precision, this research focuses exclusively on interactional justice.

Fairness Heuristic Theory (FHT) provides a framework for explaining why interactional justice amplifies rather than buffers the negative effects of LMXA (Lind, 2001). FHT assumes that employees use fairness as a heuristic to form expectations about authority behavior. When an organization demonstrates high interactional justice, employees develop strong expectations of consistent, respectful treatment from their leaders (Rogers & Ashforth, 2017). This signals that leaders are trustworthy and that interactions will be predictable.

However, when a leader presents ambivalent behavior within a high-justice context, their actions constitute a significant violation of these established expectations. Research on fairness violations indicates that when baseline fairness expectations are strong, subsequent inconsistencies are perceived as more severe and evoke stronger negative reactions (Matta et al., 2017; Holtz, 2013). The contrast between expected and actual treatment drives this amplification. In contexts where interactional justice is consistently low, employees may become accustomed to inconsistent treatment, making leader ambivalence less surprising and thus less damaging to their sense of control (Tepper et al., 2017). Conversely, in high-justice environments, the same ambivalent behavior represents a significant breach of relational norms. This distinct contrast makes the unpredictability more noticeable and psychologically threatening, amplifying the damage to workplace sense of control and ultimately intensifying emotional exhaustion. This moderated mediation pattern suggests that the protective assumption of fairness may paradoxically create conditions for greater harm when leaders behave inconsistently. This leads to the following hypothesis:

**H4.**  *Interactional justice moderates the indirect effect of LMXA on emotional exhaustion via workplace sense of control, such that the mediated relationship is stronger when interactional justice is high rather than low.*

In summary, this research develops a moderated mediation framework grounded in Fairness Heuristic Theory. LMXA is proposed to increase emotional exhaustion by undermining workplace sense of control, which functions as the key psychological mechanism connecting relational ambivalence to emotional strain. Critically, the organizational justice context shapes this pathway. Interactional justice provides relational fairness signals that raise expectations of leader consistency, thereby making employees more sensitive to ambivalent behaviors and more vulnerable to control loss. This framework advances current understanding in two ways. First, it identifies the mechanism through which relational ambivalence translates into emotional strain. Second, it reveals a boundary condition under which interactional justice amplifies rather than buffers this strain. To test the hypotheses, this research employed a two-phase data collection process detailed in the following section.

## 3. Methods

### 3.1. Samples and Procedure

Data for this research were collected via Huixiang Data, a professional online survey platform. The platform has over 1.5 million registered participants across a wide range of industries and geographical regions in China. Huixiang Data supports precise sample targeting based on hundreds of user profiles, ensuring questionnaire delivery to specific demographic groups as required by the research design. This online approach ensured collection efficiency, protected participant anonymity, and reduced potential social desirability bias (Stern et al., 2014). To incentivize participation and minimize attrition between the survey waves, monetary compensation was offered for the completion of each phase.

This research employed a two-phase, time-lagged design to reduce common method bias by temporally separating the measurement of variables (Podsakoff et al., 2012). In the first phase, questionnaires were distributed to an initial sample of 550 employees across 29 provinces, measuring LMXA, interactional justice (independent and moderating variables), and demographics. Two weeks later, these same participants were invited to complete a second survey that assessed workplace sense of control and emotional exhaustion (mediating and dependent variables). This time-lagged design helps to reduce the influence of temporary mood states and consistency motifs (Podsakoff et al., 2003). A total of 511 valid matched responses were obtained, yielding a high effective response rate of 92.9%. The diversity of samples across various organizational types enhances the generalizability of the findings (Highhouse & Gillespie, 2010).

The demographic results of the participants showed a relatively balanced and diverse sample. In terms of gender, females accounted for a slightly larger proportion (56.2%) compared with males (43.8%). Regarding age distribution, the largest group was employees aged 26–30 years (43.8%), followed by those aged 31–35 years (21.7%). Most participants were highly educated, the majority of participants holding a bachelor’s degree (75.3%). Only 12.9% had a junior college degree or below, while 10.2% had a master’s degree and 1.6% held a doctoral degree or above.

In terms of organizational types, most participants worked in private enterprises (57.5%), followed by state-owned enterprises (35.4%), and a smaller proportion in foreign or joint ventures (7%). This distribution reflects the significant role of private firms in China’s economy while still maintaining representation from other organizational forms. Job position analysis shows that frontline employees made up the majority (56.8%), with first-line managers (34.8%) and middle managers (8.4%). This suggests that the data primarily capture employees’ perceptions at the operational level. Tenure analysis reveals that 38.7% of participants had worked in their organizations for 1–3 years, and 33.7% for 4–6 years, indicating that the majority were relatively stable employees with moderate organizational experience. In contrast, only 8.2% had less than one year of tenure, and 8.2% had more than ten years. Regarding tenure with direct supervisors, the largest group reported 1–3 years (39.5%), followed by more than 3 years (30.3%). This distribution suggests that most employees had already developed working relationships with their supervisors, which provides meaningful variance for studying leader–member exchange ambivalence. Table 1 below shows the demographic analysis of this research (n = 511). Descriptive analyses were conducted using SPSS 31.0.0.0.

### 3.2. Measures

All scales used in this research were originally developed in English and underwent a rigorous translation and back-translation procedure to ensure semantic and conceptual equivalence. Two bilingual scholars first translated the items into Chinese, and two independent experts subsequently back-translated them into English. Discrepancies were discussed and resolved by experts familiar with organizational behavior constructs. This process allowed the translated instruments to retain both linguistic accuracy and cultural appropriateness.

All variables, except demographic information, were measured using a 6-point Likert scale ranging from 1 (strongly disagree) to 6 (strongly agree). The use of a 6-point format, instead of the more common 5-point or 7-point scales, was intended to reduce central tendency bias by avoiding a neutral midpoint (Douven, 2018). In addition, for online surveys, a 6-point scale could lower respondent burden during selection, especially when measuring multiple constructs in a single instrument (Jebb et al., 2021; Chyung et al., 2017). As the measured variables in this research concern subjective feelings and evaluations, such as workplace sense of control, emotional exhaustion, and perceptions of interactional justice, a 6-point scale can better capture emotional nuance by forcing participants to express a clear position rather than selecting an ambiguous middle option. This approach could improve the reliability and validity of these measures (Kusmaryono et al., 2022). Thus, the 6-point format was deemed the most appropriate for this research.

***Leader–member exchange ambivalence (LMXA)*** was assessed using the seven-item scale developed by Lee et al. (2019). This construct captures employees’ conflicting cognition and feelings toward their supervisor, such as simultaneously perceiving supportive and critical behaviors. A representative item is “I have conflicting thoughts: sometimes I think that my working relationship with my manager is very good, while at other times I don’t.” Cronbach’s α in this research was 0.88, indicating good internal consistency.

***Interactional justice*** was measured with J. A. Colquitt’s (2001) scale, which distinguishes between interpersonal justice and informational justice. Interpersonal justice refers to whether employees feel treated with dignity, politeness, and respect by their supervisor, while informational justice represents the adequacy and honesty of explanations regarding organizational procedures. By examining these two aspects, the measure provides a comprehensive assessment of fairness in social interactions at work. Sample items include “My manager treats me with respect” (interpersonal justice) and “My manager communicates details in a timely manner” (informational justice). Cronbach’s α for the overall interactional justice scale was 0.90.

***Workplace sense of control*** was measured using an adapted version of the scale developed by Lachman and Weaver (1998). The original scale assesses a general sense of control over one’s life. It consists of two parts: personal mastery, which reflects the belief that effort and persistence can lead to success; and perceived constraints, which shows beliefs about external barriers that restrict autonomy. For the purpose of this research, all items were rephrased to specifically reflect the workplace context. Sample items include “At work, I can do just about anything I really set my mind to.” (personal mastery) and “Other people at work determine most of what I can and cannot do.” (perceived constraints). To calculate the overall workplace sense of control, the perceived constraints items were reverse-scored so that higher scores consistently represent stronger control beliefs. Cronbach’s α was 0.92, suggesting excellent internal consistency.

***Emotional exhaustion*** was assessed using the five-item subscale of the Maslach Burnout Inventory-General Survey (MBI-GS) (Schaufeli et al., 1996). This scale captures feelings of being emotionally overextended and drained by one’s job, such as “I feel emotionally exhausted by my job.” Emotional exhaustion is a core dimension of burnout and is closely associated with prolonged job stress and decreased well-being. Cronbach’s α was 0.96, indicating excellent reliability.

Finally, ***demographic variables*** were included as controls, such as gender, age, education, organizational type, job position, tenure in organizations, and tenure with direct supervisor. These variables have been shown to influence fairness perceptions, stress, and leader–member relationships in prior organizational behavior research (Ma et al., 2023; González-Navarro et al., 2018; J. A. Colquitt et al., 2013b). During the data analysis phase, categorical control variables with more than two levels, specifically organization type and position, were converted into dummy variables before being entered into the regression models to ensure appropriate statistical treatment.

## 4. Results

### 4.1. CFA Analysis

Since the variables in this research (LMX ambivalence, interactional justice, workplace sense of control, and emotional exhaustion) were all assessed using multi-item scales, it was necessary to examine the construct distinctiveness before hypothesis testing. A confirmatory factor analysis (CFA) was conducted using Python 3.12.7 to validate the measurement model.

The hypothesized four-factor model demonstrated acceptable fit to the data (χ^2^ (489) = 1574.19, χ^2^/df = 3.22, CFI = 0.904, TLI = 0.896, IFI = 0.867, RMSEA = 0.066). To establish discriminant validity, this model was compared against several more parsimonious alternative models, as detailed in the notes of Table 2. As shown in Table 2, the fit of all alternative models was significantly poorer (e.g., single-factor model: χ^2^/df = 10.77, CFI = 0.571, RMSEA = 0.138). These comparisons confirm that the four constructs are empirically distinct, and the hypothesized measurement model is appropriate for subsequent analyses.

Internal consistency was satisfactory, with Cronbach’s alpha values ranging from 0.88 to 0.96. Although IFI value was slightly below the conventional 0.90 threshold, the CFI and RMSEA values and the significant improvement of fit over alternative models indicate that the measurement model is adequate for subsequent analyses.

Finally, the poor fit of the single-factor model (χ^2^/df = 10.77, CFI = 0.571, RMSEA = 0.138) serves as a statistical check for common method bias. According to Harman’s single-factor test principle, the fact that a single general factor does not account for the majority of the variance in the data suggests that CMB is unlikely to be a serious threat to the interpretation of the results (Podsakoff et al., 2003).

### 4.2. Correlation Analysis

Descriptive statistics and intercorrelations of all variables are presented in Table 3. LMX ambivalence was positively correlated with emotional exhaustion (r = 0.33, *p* < 0.01) and negatively correlated with workplace sense of control (r = −0.22, *p* < 0.01). workplace sense of control, in turn, was strongly and negatively correlated with emotional exhaustion (r = −0.68, *p* < 0.01). Interactional justice exhibited positive correlations with workplace sense of control (r = 0.55, *p* < 0.01) and negative correlations with emotional exhaustion (r = −0.41, *p* < 0.01). These patterns provide preliminary support for the hypothesized mediating and moderating effects and justify further tests.

### 4.3. Hypothesis Testing

Before testing the hypotheses, this research assessed the potential issue of multicollinearity by conducting Variance Inflation Factor (VIF) diagnostics for all predictors in the regression models. The highest VIF value was 3.05, which is well below the commonly accepted threshold of 10 (Hair et al., 2009). This indicates that multicollinearity was not a significant concern that would compromise the interpretation of the regression coefficients.

The hypotheses were tested using hierarchical regression analysis in SPSS 31.0.0.0., with the results presented in Table 4. To provide a more robust test of the overall moderated mediation model, a supplementary analysis using the PROCESS macro (Model 7) was conducted, with results shown in Table 5 and Table 6.

#### 4.3.1. Hierarchical Regression Analysis

As shown in Table 4, the results provide support for all proposed hypotheses. The detailed analysis is as follows.

**Table 4:** Results of hierarchical regression analysis.

Variable	Model 1	Model 2	Model 3	Model 4	Model 5	Model 6
	(DV: SoC)	(DV: SoC)	(DV: SoC)	(DV:EE)	(DV: EE)	(DV: EE)
	B	B	B	B	B	B
**Step 1: Control Variables**						
Gender	0.03	−0.01	0.01	−0.01	−0.01	0.01
Age	−0.03	−0.01	−0.01	0.04	−0.02	−0.02
Education	0.03	−0.01	−0.01	−0.00	0.05	0.06
Workyear	0.06	0.06	0.07	−0.19	−0.14	−0.09
Time_sup	0.13 *	0.03	0.02	−0.31 **	−0.22 *	−0.12
Org_type (State-owned vs. Private)	−0.11	−0.04	−0.04	0.13	0.07	−0.02
Org_Fore (Foreign/Joint vs. Private)	−0.13	−0.25 *	−0.26 *	0.34	0.34	0.20
Pos_F (First-line vs. Frontline)	0.15	0.08	0.07	−0.11	−0.17	0.01
Pos_M (Middle mgr. vs. Frontline)	0.22	−0.01	0.01	0.16	0.17	0.40 *
**Step 2: Main Effects**						
LMXA (centered)		−0.14 ***	−0.12 ***		0.39 ***	0.23 ***
Interactional Justice (centered)		0.62 ***	0.62 ***			
WSoC_all						−1.06 ***
**Step 3: Interaction Term**						
LMXA × Interactional Justice			−0.10 *			
R^2^	0.05	0.36	0.37	0.08	0.16	0.51
ΔR^2^	—	0.31 ***	0.01 *	—	0.08 ***	0.35 ***
F	2.93 **	25.44 ***	24.10 ***	4.88 ***	9.57 ***	47.18 ***

Notes: N = 511. Unstandardized coefficients (B) are reported. DV = Dependent Variable; WSoC = Workplace Sense of Control; EE = Emotional Exhaustion; Time_sup = Tenure with supervisor; Org_type = Organization type (state-owned vs. private enterprise as reference); Org_Fore = Organization type (foreign/joint venture vs. private enterprise as reference); Pos_F = Position (first-line manager vs. frontline employee as reference); Pos_M = Position (middle manager vs. frontline employee as reference). All analyses controlled for demographic and organizational variables as shown. * *p* < 0.05. ** *p* < 0.01. *** *p* < 0.001.

**Hypothesis 1**, which predicted a positive relationship between LMXA and emotional exhaustion, was supported. Model 5 shows that after accounting for control variables, LMXA was a significant positive predictor of emotional exhaustion (B = 0.39, *p* < 0.001).

**Hypothesis 2** proposed that LMXA would be negatively related to workplace sense of control. This was also supported. In Model 2, LMXA significantly negatively predicted workplace sense of control (B = −0.14, *p* < 0.001).

**Hypothesis 3** suggested that workplace sense of control mediates the relationship between LMXA and emotional exhaustion. To test this, this research examined Model 6. The results show that when workplace sense of control was included in the model, its effect on emotional exhaustion was significant (B = −1.06, *p* < 0.001). Importantly, the direct effect of LMXA on emotional exhaustion decreased (from B = 0.39 in Model 5 to B = 0.23 in Model 6) but remained significant. This pattern confirms that workplace sense of control acts as a partial mediator, supporting Hypothesis 3.

Finally, **Hypothesis 4** predicted that interactional justice would moderate the relationship between LMXA and workplace sense of control. Model 3 tests this interaction. The interaction term (LMXA × Interactional Justice) was significant (B = −0.10, *p* < 0.05), and its inclusion explained a significant additional 1% of the variance in workplace sense of control (ΔR^2^ = 0.01, *p* < 0.05). This result supports Hypothesis 4.

#### 4.3.2. Supplementary Analysis: Moderated Mediation Test

To supplement the hierarchical regression, this research applied the PROCESS macro (Model 7) to formally test the conditional indirect effect. This analysis provides a more robust, integrated test of the overall moderated mediation model. The results are presented in Table 5 and Table 6.

Table 5 displays the unstandardized regression coefficients for the paths within the model. Consistent with the previous analysis, the interaction term between LMXA and interactional justice significantly predicted workplace sense of control (B = −0.10, *p* < 0.05), reaffirming the moderating effect.

**Table 5:** Results of moderated mediation analysis (PROCESS Model 7).

	Coeff.	SE	t	*p*	LLCI	ULCI
**Mediator Model (** **Outcome: Workplace Sense of Control)**						
LMXA (X)	−0.12 ***	0.03	−3.98	<0.001	−0.179	−0.061
Interactional Justice (W)	0.62 ***	0.04	14.67	<0.001	0.540	0.707
LMXA × Interactional Justice (X×W)	−0.10 *	0.04	−2.52	0.012	−0.183	−0.023
R^2^ = 0.367 (ΔR^2^ = 0.008)						
**Dependent Variable Model (Outcome: Emotional Exhaustion)**						
Workplace Sense of Control (M)	−1.06 ***	0.06	−18.85	<0.001	−1.167	−0.947
LMXA (Direct Effect, X)	0.23 ***	0.04	5.20	<0.001	0.142	0.315
R^2^ = 0.510						

Notes: N = 511. Bootstrap sample size = 5000. Coeff. = unstandardized coefficient; SE = standard error; CI = confidence interval; LLCI = lower-level confidence interval; ULCI = upper-level confidence interval. All analyses controlled for gender, age, education, organizational tenure, tenure with supervisor, and dummy-coded variables for organization type and position. * *p* < 0.05. *** *p* < 0.001.

Table 6 presents the core findings of the moderated mediation analysis. The results revealed a significant **index of moderated mediation** (Index = 0.109, 95% CI [0.025, 0.199]). Because the confidence interval does not contain zero, this confirms that the indirect effect of LMXA on emotional exhaustion through workplace sense of control is significantly moderated by interactional justice.

**Table 6:** Conditional Indirect Effects and Index of Moderated Mediation.

**Conditional Indirect Effects at Values of Interactional Justice**				
Interactional Justice Level	Effect	BootSE	BootLLCI	BootULCI
Low (−1 SD = −0.72)	0.048	0.055	−0.064	0.151
Mean (0.00)	0.145	0.030	0.086	0.205
High (+1 SD = +0.72)	0.206	0.034	0.138	0.273
**Index of Moderated Mediation**				
Moderator	Index	BootSE	BootLLCI	BootULCI
Interactional Justice	0.109	0.044	0.025	0.199

Notes: N = 511. Bootstrap sample size = 5000. SE = standard error; CI = confidence interval; Boot = bootstrap; LLCI = lower-level confidence interval; ULCI = upper-level confidence interval. All analyses controlled for gender, age, education, organizational tenure, tenure with supervisor, and dummy-coded variables for organization type and position. For total indirect effect, conditional indirect effects, and index of moderated mediation, statistical significance is determined by 95% bootstrap confidence intervals that do not contain zero.

**Figure 2:**
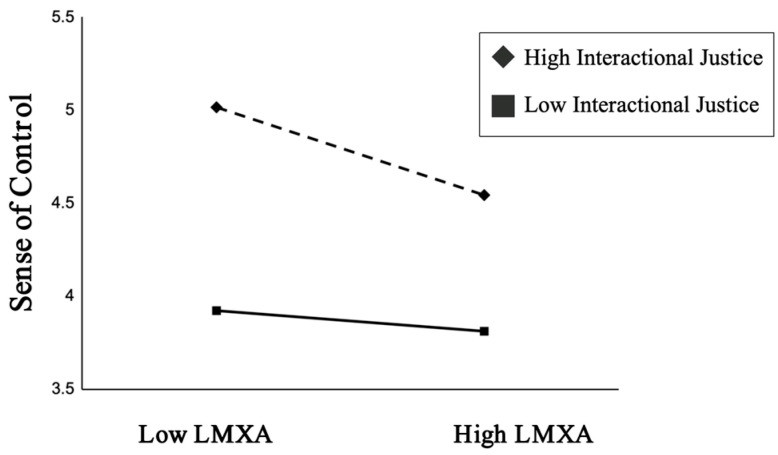
Moderating effect of interactional justice. Notes: The figure plots the simple slopes of the relationship between LMXA and workplace sense of control at high and low levels of interactional justice. The negative association is stronger at high levels of interactional justice.

An examination of the conditional indirect effects further clarifies this relationship. The indirect effect was not significant at low levels of interactional justice (Effect = 0.048, 95% CI [−0.064, 0.151]) but was significant and positive at mean (Effect = 0.145) and high levels (Effect = 0.206) of interactional justice. This provides strong support for the research hypothesis, demonstrating that the negative, indirect effect of LMXA on emotional exhaustion (via diminished workplace sense of control) is stronger when employees perceive higher levels of interactional justice. A plot of this interaction is provided in Figure 2.

## 5. Discussion

The findings reveal that LMX ambivalence significantly increases employee emotional exhaustion, and that this effect is partially mediated by reduced workplace sense of control. Moreover, this research examined the boundary conditions under which fairness may intensify the strain of ambivalence. The results show that interactional justice strengthens the indirect effect between LMXA and the workplace sense of control. The pattern indicates that fairness signals can magnify the strain caused by relational ambivalence, challenging assumptions about the universally beneficial nature of justice. The following sections elaborate on the theoretical and practical implications of this research, as well as the limitations and directions for future research.

### 5.1. Theoretical Implications

This research makes several theoretical contributions. By developing an integrated model grounded in Fairness Heuristic Theory (FHT), this research clarifies the complex interaction between LMXA, the justice context, and employee well-being, offering novel insights into these domains.

First, this research examines leader–member exchange ambivalence (LMXA) as a negative workplace stressor. Previous research on leader–member relations investigated the relationship on a simple scale from high quality to low quality (continuum view of LMX quality) (Lee et al., 2019). Lee et al. (2019) introduced LMXA, which depicts that leader and follower may experience mixed and conflicting feelings during interactions. Several researchers have investigated that its inconsistency nature may cause negative outcomes, such as job anxiety (Huang et al., 2022), ego depletion (Liu & Liu, 2024), and diminished voice behavior (Leong et al., 2026; Liu & Liu, 2024). This research also confirms a strong, positive link between LMXA and employee emotional exhaustion (B = 0.23, *p* < 0.001), extending the work of Lee et al. (2019) and Han and Sears (2024). This result supports the idea that an ambivalent relationship is not just a midpoint between positive and negative. It aligns with Rothman and Melwani’s (2017) work, which suggests that dealing with mixed signals requires significant mental energy and depletes an employee’s psychological resources. Therefore, this research shows why LMXA is a critical topic in leader–member relationships.

Second, this research contributes to the LMXA literature by identifying workplace sense of control as a crucial psychological mechanism between LMXA and emotional exhaustion. Recent studies have examined various mediating mechanisms linking LMXA to employee outcomes. For instance, Han and Sears (2024) demonstrated that LMXA reduces work engagement and increases emotional exhaustion through need frustration, while T. Chen et al. (2025) found that diminished relational energy mediates the relationship between LMXA and counterproductive work behavior. However, these studies have focused primarily on motivational or affective mechanisms. This research extends existing literature by demonstrating that the inherent unpredictability of LMXA directly damages employees’ sense of control. This finding is critical as loss of control is a significant pathway through which various workplace stressors are converted into psychological strain (Nauman et al., 2020; Spector, 1986). By empirically linking LMXA to this fundamental psychological resource, this research provides a clear process model of its detrimental effects.

Third, this research contributes to the organizational justice literature by challenging the conventional assumption that fairness universally benefits employees. Previous research has established justice as a protective resource that helps employees manage workplace demands (J. A. Colquitt et al., 2013b). However, this research reveals a paradoxical pattern. The indirect effect was nonsignificant at low interactional justice but significant at high levels (Effect = 0.206, 95% CI [0.138, 0.273]). The finding that high interactional justice amplifies LMXA’s negative effect aligns with emerging research on paradoxical outcomes of positive organizational contexts. For instance, Matta et al. (2017) found that inconsistent fair treatment could be more stressful than consistently unfair treatment. Similarly, research on expectancy violations has shown that positive contexts could intensify negative reactions when expectations are breached (Adamovic, 2023; Matta et al., 2017). The null effect at low justice and significant effect at high justice directly supports the contrast mechanism. When justice is low, employees lack established expectations to be violated, making leader ambivalence less psychologically threatening. When justice is high, the same behavior constitutes a salient violation that amplifies harm. This contributes a significant boundary condition to understanding when and why fairness may fail to protect employees.

Fourth, this research extends Fairness Heuristic Theory (FHT) by demonstrating a critical contingency for its application. The core assumption of FHT is that employees use fairness as a heuristic to manage uncertainty and form positive expectations about authorities (Van den Bos, 2005). Most research has focused on the positive outcomes of this process, such as increased trust and cooperation (Cropanzano et al., 2017; J. A. Colquitt et al., 2013b). This research reveals the negative consequences when expectations are violated. The findings support the statement of Qin et al. (2015), who argued that employees are more sensitive to inconsistent signals when their general fairness perceptions are strong. By linking expectation violation to LMXA and its outcome mechanism (loss of control leading to exhaustion), this research reveals a critical limitation of the fairness heuristic. Its positive effects can reverse into amplified harm when leader behavior contradicts established fairness expectations.

Finally, this research offers an integrated contribution by highlighting the importance of alignment between organizational justice and leadership behavior. The findings move beyond static models that treat LMXA and justice as independent effects to reveal their dynamic interactions. This aligns with Y. Zhang et al. (2014), who emphasized the need to examine how justice and leadership mutually influence employee outcomes. The key insight is that organizations cannot simply cultivate fair systems and expect universal benefits. When organizational contexts promote high interactional justice but leaders behave ambivalently in daily interactions, this misalignment creates greater harm than consistent low justice environments. These findings have important implications for practice, and the following section discusses how organizations can apply these insights.

### 5.2. Practical Implications

The findings of this research generate important implications for leadership development, organizational justice management, and employee well-being interventions.

First, the results highlight the practical need for organizations to address ambivalent leader–member exchanges. Ambivalence in supervisory relationships is usually overlooked, as leaders may believe that occasional positive signals balance negative ones (Herr et al., 2019). Particularly when leaders behave differently under routine versus stressful conditions, leadership inconsistency creates substantial strain for both leaders and followers (Klebe et al., 2022). This research shows that such inconsistency undermines employees’ control perceptions and increases emotional exhaustion. Leaders therefore need training to maintain behavioral consistency and reduce mixed signals. Therefore, leadership development programs should shift their focus from merely encouraging positive behaviors to cultivating behavioral consistency. To address this issue, firstly, HR teams should establish a structured feedback protocol where leaders schedule regular, predictable check-ins with each team member at consistent intervals. The structured protocol could reduce uncertainty and allow employees to anticipate when they will receive guidance. Secondly, organizations should implement a decision-making transparency practice where leaders explain the rationale behind their decisions. It could help employees understand why certain choices were made, even unfavorable decisions become more predictable and less ambiguous. Furthermore, organizations should provide leaders with sufficient stress coping resources, which facilitates leaders to maintain consistency during high-pressure periods. Thirdly, leaders should create self-monitoring tools, such as weekly reflection dairy where leaders assess whether their actions aligned with their stated expectations. This helps leaders identify unintentional inconsistencies.

Second, the identification of the workplace sense of control as a mediating mechanism emphasizes the importance of enhancing employees’ autonomy and decision independence in practice. Recent reviews confirm that perceived control plays as a significant personal resource for employee well-being, enabling individuals to cope with stressors (Dollard & Bailey, 2021; Ganster & Rosen, 2013). Even when faced with relational ambivalence, organizations could mitigate its harmful effects by proactively strengthening employees’ sense of control. F. Zhang and Parker (2022) suggest that perceived autonomy support from organizations predicts increased job crafting behaviors, which in turn enhance workplace well-being. Therefore, organizations should implement goal-setting sessions where employees collaborate with their supervisors to define working objectives rather than being assigned with tasks. During these processes, employees would know how goals will be achieved, what resources are needed, and what indicators will define success. Furthermore, organizations should provide employees with choice in how they accomplish their work, such as flexibility in work schedules, work location, or the sequence in which tasks are completed. Even small choices can significantly enhance employee workplace sense of control.

Third, and most critically, this research suggests a more nuanced approach to managing organizational justice, challenging the traditional view that a leader’s responsibility is limited to ensuring organizational justice (J. A. Colquitt et al., 2013a). This research reveals that promoting high interactional justice creates expectations that can backfire when leadership behavior is inconsistent. The findings serve as a clear warning that high-justice cultures amplify harm when leaders behave ambivalently. The key practical implication is not to build fair systems but to ensure alignment between formal justice systems and the daily relational practices of leaders. To achieve this, organizations should firstly recognize that heavily promoting fairness values through public commitments creates heightened employee expectations. Before launching such initiatives, organizations should assess leadership behavior before raising justice expectations. Secondly, an alignment monitoring system, such as quarterly surveys or 360-dgree feedback, should be adopted to ask employees, peers, and supervisors whether the leader treats people fairly across different situations and over time. Thirdly, during organizational change periods or when promoting new managers, organizations should practice expectation management by transparently acknowledging that consistency may be challenging during transitions. Fourthly, the HR team should create peer support networks or employee resource groups where individuals can share experiences and develop collective coping strategies when they encounter leadership inconsistencies. Finally, for organizations with established high organizational justice, it is necessary to arrange regular leadership calibration meetings where leaders discuss specific cases to ensure they are applying fairness standards consistently across teams. This prevents situations where one leader’s ambivalent behavior undermines the credibility of the entire organizational justice system.

Finally, the findings suggest interventions focused on employee well-being. As relational ambivalence and fairness violations drain psychological resources, organizations should provide support systems to enhance employees’ coping capacities. Evidence suggests that workplace social support could buffer the harmful effects of stressors such as job overload (Jolly et al., 2021). Similarly, Edmondson and Lei (2014) stated that psychological safety enables employees to express concerns and seek help without fear of negative consequences. Although direct evidence is limited, creating such a supportive climate could provide employees with a protective environment to reduce exhaustion and enhance resilience. To operationalize these support systems, organizations could implement the following practices. First, establish formal mentoring programs that pair employees with trusted colleagues outside their teams, providing a safe space to discuss relational challenges with their supervisors. Second, train team members to recognize signs of emotional exhaustion in their colleagues and create norms where checking in on each other’s well-being is valued rather than stigmatized. Third, create assistance programs that include confidential counseling services addressing workplace relationship stress. Fourth, train leaders to give non-defensive feedback when employees raise concerns about inconsistent treatment, also reward employees who speak up. Fifth, implement regular team reflective sessions where employees can collectively discuss what is working and what is not in their work environment, creating a culture of continuous improvement.

These implications emphasize that organizations should not solely rely on fairness practices to guarantee positive outcomes. Instead, they should address the alignment between organizational justice and leader behaviors, strengthen employees’ workplace sense of control through structured autonomy-enhancing practices, and provide a supportive environment through accessible support systems. By attending to these system-level factors with specific, actionable strategies, organizations can translate fairness into tangible improvements in employee well-being rather than unintentionally amplifying the strain of ambivalence.

### 5.3. Limitations and Future Directions

While this research offers new insights into the complex interplay between leader–member exchange ambivalence (LMXA) and organizational justice, several limitations should be noted for future studies. First, although this research employed a two-phase data collection design to mitigate common method bias, all data were derived from employee self-reports. This approach may risk increasing the relationships between variables due to the single-source nature of the data (Podsakoff et al., 2012). Future studies could enhance the robustness of the findings by incorporating multi-source data. For instance, leaders could be invited to assess the quality of the leader–member relationship, or peers could evaluate an employee’s level of emotional exhaustion, thereby providing more objective measures (Conway & Lance, 2010). Furthermore, the cross-sectional design of this research constrains the ability to deliver causal relationships. While the theoretical model proposes a path from LMXA to emotional exhaustion, the reverse causality might also exist. For instance, emotionally exhausted employees might be more inclined to interpret their leader’s behavior as ambivalent and inconsistent. Therefore, future research could adopt a longitudinal design, tracking the variables at multiple time points to test the causal relationships (Ployhart & Vandenberg, 2010).

Second, this research lacks attention to the potential influence of dispositional traits. Constructs such as LMX ambivalence and emotional exhaustion are affective in nature and may be influenced by stable individual differences like positive or negative affectivity (Kaplan et al., 2009). For example, individuals high in negative affectivity tend to experience distressing emotions and may tend to interpret workplace events, including leader interactions, through a more negative lens (Rosen et al., 2016). This raises the possibility that the relationship between LMXA and emotional exhaustion could be partially attributable to such traits rather than solely to the relational dynamics. Although the time-lagged design helps to reduce common method bias (Podsakoff et al., 2003), future research would benefit from directly measuring and including dispositional variables as controls. This would allow for a more rigorous test of the model by investigating the effects of relational stressors from individual tendencies.

Third, the sample for this research was drawn exclusively from the Chinese organizational context. Cultural values, such as power distance and collectivism, may influence how employees perceive and react to leader behaviors and organizational justice (Hofstede, 2001). For instance, in cultures with high power distance, employees might present greater tolerance for inconsistencies in leadership behavior, which could potentially weaken the negative effects of LMXA. Consequently, it remains unclear whether the findings of this research could be generalized to other cultural settings, particularly western individualistic cultures. Future research could conduct cross-cultural comparisons to explore the moderating role of cultural values in the relationship between LMXA and its outcomes, thereby testing the applicability of the research model across diverse cultures (Taras et al., 2010).

Fourth, this research identifies workplace sense of control as a key psychological mechanism connecting LMXA to emotional exhaustion, yet it is not the sole explanatory pathway. The relational uncertainty caused by LMXA could also affect employee well-being through other mechanisms. For instance, employees may perceive ambivalent leader behavior as psychological contract breach. They feel leaders fail to provide promised stability and support, triggering negative emotional outcomes (Balogun et al., 2018). Additionally, when employees try to understand leaders’ conflicting behaviors, they may deplete mental energy and experience internal conflict between different thoughts and expectations. This mental strain might challenge employees who have a high need for cognitive closure, i.e., those who prefer clear, definitive answers rather than ambiguity (Kruglanski et al., 2018). Such cognitive processing may create psychological stress and discomfort (T. Chen et al., 2025; Baldner et al., 2022). Future research should examine these psychological processes to better understand how inconsistent leadership affects employees.

Fifth, this research focuses on individual-level outcomes overlooks potential team and organizational consequences of ambivalent leader–member relationships. LMXA may produce broader effects influencing team cohesion, collective efficacy, and organizational climate (Ciampa et al., 2021; Jansen et al., 2016). Future research should examine these higher-level outcomes to understand broader organizational implications of leadership ambivalence. Furthermore, the static model of ambivalent relationships fails to capture the dynamic evolution between leaders and followers. Longitudinal investigations could examine how LMXA patterns change across different interaction contexts and whether certain organizational supports could facilitate, stabilize or improve ambivalent relationships over time.

Finally, the exclusive focus on emotional strain may provide an incomplete picture of LMXA consequences. Ambivalent relationships, despite their challenges, might promote employee learning, adaptability, or resilience (Luthans et al., 2014; Edmondson & Lei, 2014). Future research should adopt a more balanced perspective, investigating when and how ambivalent relationships might deliver positive outcomes. These limitations suggest opportunities for future research. Such studies could advance understanding of ambivalent relationships and provide practical guidance for leaders and organizations.

## 6. Conclusions

This research investigated how leader–member exchange ambivalence (LMXA) influences employee emotional exhaustion through the mediating role of workplace sense of control, and examined the moderating influence of organizational justice, especially interactional justice, on this process. Employing Fairness Heuristic Theory, this research proposed a model suggesting that in a high-justice environment, the detrimental effects of LMXA would be amplified due to expectation violation.

This research makes a primary theoretical contribution by uncovering the paradoxical nature of organizational justice. Challenging the conventional perspective that fairness is uniformly beneficial, this research demonstrates that high justice could create strong expectations of consistency. When these expectations are violated by an ambivalent leader, the psychological drain for employees is magnified. This finding advances a more nuanced understanding of how justice operates, highlighting that its effects are contingent on the alignment between established expectations and daily experiences. From a practical perspective, this research highlights the importance of fair systems and consistent leadership in organizations. When organizations empower employees with greater control, they could significantly reduce relational stress and improve overall workplace well-being.

## Figures and Tables

**Table 1:** Demographic analysis (n = 511).

Variable	Category	Frequency (N)	Percentage (%)
Gender	Male	224	43.8
	Female	287	56.2
Age	≤25 years	99	19.4
	26–30 years	224	43.8
	31–35 years	111	21.7
	36–40 years	43	8.4
	≥41 years	34	6.7
Education	Junior college or below	66	12.9
	Bachelor’s degree	385	75.3
	Master’s degree	52	10.2
	Doctoral degree or above	8	1.6
Organization Type	State-owned enterprise	181	35.4
	Private enterprise	294	57.5
	Foreign/Joint venture	36	7
Position	Frontline employee	290	56.8
	First-line manager	178	34.8
	Middle manager	43	8.4
Tenure in Org.	<1 year	42	8.2
	1–3 years	198	38.7
	4–6 years	172	33.7
	7–10 years	57	11.2
	>10 years	42	8.2
Tenure with Sup.	<0.5 year	32	6.3
	0.5–1 year	122	23.9
	1–3 years	202	39.5
	>3 years	155	30.3

**Table 2:** Confirmatory factor analysis results comparing alternative measurement models.

Model	χ^2^	df	χ^2^/df	IFI	TLI	CFI	RMSEA
*Hypothesized Model*							
Four-factor model	1574.19	489	3.22	0.867	0.896	0.904	0.066
*Alternative Models*							
Three-factor model	2931.42	492	5.96	0.752	0.768	0.784	0.099
Two-factor model	4362.29	494	8.83	0.630	0.633	0.657	0.124
Single-factor model	5332.10	495	10.77	0.548	0.542	0.571	0.138

Notes: N = 511. χ^2^ = Chi-square; df = degrees of freedom; RMSEA = Root mean square error of approximation; IFI = Incremental fit index; TLI = Tucker–Lewis index; CFI = comparative fit index. The hypothesized four-factor model included LMX ambivalence, interactional justice, workplace sense of control, and emotional exhaustion as distinct factors. Alternative models were tested for comparison: Three-factor model: LMXA and emotional exhaustion were combined into a single “strains” factor. Two-factor model: the “strains” factor was retained, and interactional justice and workplace sense of control were combined into a single “resources” factor. Single-factor model: all items were loaded onto a single general factor.

**Table 3:** Means, standard deviations, reliabilities, and correlations among variables.

Variable	M	SD	1	2	3	4	5	6	7	8	9	10	11
1. Gender	1.56	0.50	—										
2. Age	2.39	1.09	−0.15 **	—									
3. Education	2.00	0.54	−0.02	−0.03	—								
4. Organization type	1.72	0.59	−0.01	−0.01	−0.08	—							
5. Position	1.52	0.65	−0.14 **	0.33 **	0.14 **	0.00	—						
6. Tenure in org.	2.72	1.04	−0.18 **	0.71 **	0.02	−0.01	0.35 **	—					
7. Tenure with sup.	2.94	0.89	−0.11 *	0.49 **	−0.02	0.08	0.24 **	0.68 **	—				
8. LMXA	3.87	1.10	0.02	−0.02	−0.06	−0.06	−0.02	−0.14 **	−0.20 **	(0.88)			
9. Inte_jus	4.72	0.74	0.00	0.09	0.04	0.09 *	0.17 **	0.10 *	0.14 **	−0.05	(0.90)		
10. EE	3.52	1.47	0.03	−0.15 **	0.01	−0.02	−0.08	−0.23 **	−0.26 **	0.33 **	−0.41 **	(0.96)	
11. WSoC	4.31	0.86	−0.02	0.11 *	0.02	0.04	0.14 **	0.17 **	0.19 **	−0.22 **	0.55 **	−0.68 **	(0.92)

Notes: N = 511. M = Mean; SD = Standard Deviation. Gender was coded as 1 = Male, 2 = Female. Reliabilities (Cronbach’s α) appear on the diagonal in parentheses. Tenure in org. = Tenure in organizations; Tenure with sup. = Tenure with the direct supervisor; LMXA = Leader–Member Exchange Ambivalence; Inte_jus = Interactional Justice; EE = Emotional Exhaustion; WSoC = Workplace Sense of Control. * *p* < 0.05. ** *p* < 0.01. (2-tailed).

## Data Availability

The data supporting the findings of this study can be obtained from the corresponding author upon justified request and subject to approval.
